# African swine fever virus enhances viral replication by increasing intracellular reduced glutathione levels, which suppresses stress granule formation

**DOI:** 10.1186/s13567-024-01433-4

**Published:** 2024-12-20

**Authors:** Han Gao, Taoming Gu, Xiaopeng Gao, Zebu Song, Jing Liu, Yi Song, Guihong Zhang, Yankuo Sun

**Affiliations:** 1 African Swine Fever Regional Laboratory of China (Guangzhou), South China Agricultural University, Guangzhou, 510642 China; 2 College of Veterinary Medicine, South China Agricultural University, Guangzhou, 510642 China; 3 Guangdong Provincial Key Laboratory of Zoonosis Prevention and Control, College of Veterinary Medicine, South China Agricultural University, Guangzhou, 510642 China; 4 Maoming Branch, Guangdong Laboratory for Lingnan Modern Agriculture, Maoming, 525000 China; 5 School of Animal Science and Technology, Foshan University, Foshan, 528225 China; 6 Guangdong Provincial Key Laboratory of Animal Molecular Design and Precise Breeding, College of Animal Science and Technology, Foshan University, Foshan, 528225 China

**Keywords:** African swine fever virus (ASFV), stress granules (SGs), reduced glutathione (GSH)

## Abstract

African swine fever virus (ASFV) is a DNA virus that has significantly impacted the global swine industry. Currently, there are no effective therapies or vaccines against ASFV. Stress granules (SGs), known for their antiviral properties, are not induced during ASFV infection, even though reactive oxygen species (ROS) are generated. The mechanism by which ASFV regulates SGs formation remains unclear. This study demonstrates that ASFV antagonises SGs formation and increases intracellular levels of reduced glutathione (GSH) levels. The use of the GSH inhibitor BSO and the activator NAC confirmed that the ASFV-induced increase in GSH helps to suppress SGs formation and influences viral replication. Additionally, this study revealed that ASFV enhances GSH by upregulating the antioxidant transcription factor NRF2, as well as factors involved in GSH synthesis and regeneration, such as GCLC, and those related to the ferroptosis pathway, such as SLC7A11. Furthermore, the study uncovered that ASFV manipulates intracellular GSH levels by activating the mitochondrial protein AIFM1. This regulatory mechanism helps the virus inhibit the formation of intracellular SGs, thereby creating an optimal environment for viral replication. These findings provide new insights into the molecular strategies employed by ASFV.

**Supplementary Information:**

The online version contains supplementary material available at 10.1186/s13567-024-01433-4.

## Introduction

The African swine fever virus (ASFV) belongs to the family *Asfarviridae* and the order *Asfuvirales,* and it is classified as a member of the nucleo-cytoplasmic large DNA viruses. ASFV is the causative agent of African swine fever (ASF) outbreaks in pig populations [1, 2].

Characterised by a large, complex, double-stranded DNA genome of approximately 190 kbps, ASFV encodes more than 150 open reading frames [3, 4]. The virus was first identified in Kenya in 1921 and has since spread to various regions worldwide, including Europe, the Americas, and Asia.

ASFV can be transmitted through multiple routes, including vector-borne transmission (such as through soft ticks), the ingestion of contaminated pork products, and direct or indirect contact with infected pigs [5, 6]. The clinical manifestations of ASFV infection can vary widely in virulence, ranging from mild to highly virulent forms [7–13].

This variability is primarily due to the genetic diversity among different viral strains. Infection with highly pathogenic strains of ASFV can result in severe clinical symptoms, including high fever, skin haemorrhages, and respiratory distress, with mortality rates reaching up to 100%.

Since the emergence of ASFV in China in 2018, the virus has caused significant economic repercussions for the domestic swine industry. ASFV outbreaks have significantly impacted global pork production, leading to substantial economic losses and heightened concerns regarding agricultural and food security [1, 5, 14, 15].

To counter various forms of stress, including sodium arsenite (Ars), cells can activate eIF2α kinases. This activation leads to the phosphorylation of eIF2α, which reduces global cellular protein translation efficiency and promotes the formation of stress granules (SGs) [16–20]. SGs are membrane-less cytoplasmic structures consisting of translationally stalled mRNA, translation initiation factors, and various mRNA-binding proteins. They help cells adapt to environmental stress and play a crucial role in gene expression and homeostasis.

A core component of SGs is the Ras-GTPase-activating protein (SH3 domain)-binding protein 1 (G3BP1), which acts as a tunable switch that triggers phase separation, leading to SG assembly [21]. G3BP1 also functions as an antiviral RNA-binding protein [22]. It can bind to RIG-I and viral dsRNA to enhance the interferon response [23]. For example, infection with the porcine hemagglutinating encephalomyelitis virus promotes the formation of SGs while negatively regulating viral replication [24]. However, many viruses exploit various mechanisms to regulate G3BP1 expression to inhibit the formation of stress granules and evade the immune response. For instance, the porcine enteric diarrhoea virus activates caspase-8, which cleaves G3BP1, thereby preventing SGs formation [25].

Additionally, proteins encoded by the foot-and-mouth disease virus, such as L^pro^ and the 3A protein, can specifically cleave G3BP1 to avoid host immunity mediated by SGs [26, 27]. Feline calicivirus also exhibits inhibition of G3BP1 through its NS6^pro^ protein [28]. In the case of ASFV, the viral gene DP71L inhibits SGs formation, although the DP71L-knockout strain did not show significant differences from the wild-type strain [29–31].

Our previous research indicated that ASFV retains a strong capacity for dephosphorylating eIF2α independent of DP71L, which can inhibit the formation of SGs [32]. Furthermore, a recent study identified another ASFV-encoded factor as involved in inhibiting SGs formation through G3BP1 cleavage [33]. Nevertheless, the complete mechanism by which ASFV suppresses SGs formation remains unclear.

Viruses can induce the production of intracellular reactive oxygen species (ROS) [34–36]. Previous studies have shown that ASFV infection activates ROS production in cells [37]. The generation of ROS can lead to the phosphorylation of eIF2α, which in turn triggers the formation of SGs. ROS includes free radicals, such as superoxide anions and hydroxyl radicals, and non-free radical forms, like hydrogen peroxide and singlet oxygen. When ROS accumulates excessively, it can cause oxidative stress and damage cellular components [38–40]. However, ASFV does not promote the formation of SGs despite initiating ROS production. Antioxidative pathways, particularly those involving reduced glutathione (GSH), a crucial intracellular antioxidant, help mitigate the increase in ROS levels by neutralising them. This process protects cells from oxidative stress and maintains the redox balance [40–45]. In this research, we confirm that ASFV infection inhibits the formation of SGs, including those induced by arsenic (Ars). We also investigate how ASFV counteracts SGs formation to evade the immune response and enhance viral replication by increasing intracellular GSH levels.

## Materials and methods

### Ethics statement

In this study, all infection experiments involving live ASFV were conducted in accordance with the approved standard operating procedures at the Animal Biosafety Level-3 facility of the College of Veterinary Medicine, South China Agricultural University. No animal studies were carried out in this study.

### Virus, cell lines, antibodies, and chemicals

The virus strain used in this study was ASFV GZ201801_2 (GenBank accession number ON263123), which represents a highly pathogenic strain isolated from clinical specimens during the early ASF outbreaks in 2018 [13]. Additionally, lab-preserved ASFV YNFN202103 (GenBank accession number ON400500) and the ASFV genotype I strain OURT88/3 (GenBank Accession Number AM712240) were also used in this study.

Porcine pulmonary alveolar macrophages (PAMs) were isolated from bronchoalveolar lavage fluid (BALF) for this study. In summary, the lungs of specific-pathogens-free pigs were entirely extracted after euthanasia. Sterile PBS containing antibiotics was instilled into the lungs via the trachea, and the BALF was subsequently collected. PAMs were obtained through centrifugal enrichment at 4 °C. After counting the cells, the enriched PAMs were aliquoted, frozen, and stored in a −80 °C freezer (#C40050, NCM Biotech). The PAMs were cultured in RPMI-1640 medium (#C11875-500CP, Gibco) supplemented with 10% foetal bovine serum (FBS, #C04001-500, VivaCell) and 1% penicillin–streptomycin-amphotericin B (#C100C8, NCM Biotech).

The anti-p30 mouse monoclonal antibody was produced in-house previously [46]. Antibodies against the following proteins were purchased from their respective suppliers: β-actin (#66,009–1-Ig, Proteintech), G3BP1 (#A3968, Abclonal), GCLC (#A1038, Abclonal), GPX1 (#A1110, Abclonal), GPX4 (#67,763–1-Ig, Proteintech), SOD2 (#A19576, Abclonal), NRF2 (#16,396–1-AP, Proteintech), SLC7A11 (#A2413, Abclonal), and SLC3A2 (#A3658, Abclonal), AIFM1 (#17,984–1-AP, Proteintech), and AIFM2 (#A12128, Abclonal). Thioltracker (#T10096) was purchased from Thermo Fisher Scientific, while sodium arsenite (Ars) (#S7400) was obtained from Sigma-Aldrich. L-Buthionine-(S, R)-sulfoximine (BSO, #HY-106376A) and acetylcysteine (NAC, #HY-B2015) were source from MedChemExpress (MCE).

### Transfection of small interfering RNA (siRNA)

Three si-pG3BP1/pAIFM1 sequences were designed and synthesised (Table 1). PAMs were seeded and cultured until they adhered to the culture plate. Subsequently, 100 pmol of siRNA targeting pG3BP1/pAIFM1 or si-NC was diluted in 250 μL of Opti-MEM (#31,985–062, Gibco). In a separate tube, 10 μL of Lipofectamine 3000 reagent (#L3000001, Thermo Fisher) was diluted in 250 μL of Opti-MEM. After incubating both mixtures at room temperature (RT) for 5 min, they were thoroughly mixed and allowed to incubate for an additional 15 min at RT. The final mixture was then added dropwise to the PAM culture medium. After 24 h of siRNA knockdown, the PAMs were prepared for subsequent virus inoculation.

**Table 1:** **si-RNA sequences targeting pG3BP1/pAIFM1**

name	Target sequence	Sense strand (5’-3’)	Antisense strand (5’-3’)
si-pG3BP1-1	GGACAAGUUAGAGCUUAAA	GGACAAGUUAGAGCUUAAA(dT)(dT)	UUUAAGCUCUAACUUGUCC(dT)(dT)
si-pG3BP1-2	GCAAGAACCUGUAUCUGAA	GCAAGAACCUGUAUCUGAA(dT)(dT)	UUCAGAUACAGGUUCUUGC(dT)(dT)
si-pG3BP1-3	GAUGCAGUCUAUGGACAAA	GAUGCAGUCUAUGGACAAA(dT)(dT)	UUUGUCCAUAGACUGCAUC(dT)(dT)
si-pAIFM1-1	GAUGAUCCAAAUGUCACAA	GAUGAUCCAAAUGUCACAA(dT)(dT)	UUGUGACAUUUGGAUCAUC(dT)(dT)
si-pAIFM1-2	GAGGUGAAGAGUAGAACAA	GAGGUGAAGAGUAGAACAA(dT)(dT)	UUGUUCUACUCUUCACCUC(dT)(dT)
si-pAIFM1-3	CCGCAUGUUUCUACGAUAU	CCGCAUGUUUCUACGAUAU(dT)(dT)	AUAUCGUAGAAACAUGCGG(dT)(dT)

### Virus inoculation assay

PAMs were seeded into cell culture plates and incubated at 37 ℃ with 5% CO_2_ overnight to allow for adherence. The PAMs were then washed once with RPMI-1640 and subsequently incubated with either RPMI-1640 (mock-infected) or RPMI-1640-diluted virus (ASFV-infected). After 2 h of incubation at 37 ℃ with 5% CO_2_ for 2 h, the supernatant was discarded and replaced with fresh RPMI-1640 containing 10% FBS, marking the time point of 0 h post-inoculation (hpi).

### Real-time quantitative PCR (RT-qPCR)

The samples were processed using the indicated treatments. Subsequently, RT-qPCR was conducted following the manufacturer’s instructions (#RM401 and #Q511, Vazyme). The primers targeting ASFV *cp204l* for evaluating ASFV replication are as follows: *cp204l-F-*CAGGCTCAAGAAGAATGG, *cp204l-R-*CGTTTCAAAGGAGGATGT.

### Western blot (WB)

Whole-cell lysates from mock-infected and ASFV-infected PAMs (MOI = 1) were prepared at various time points after inoculation. The lysates were obtained by mixing the samples with WB lysis buffer (#P0013J, Beyotime) containing a cocktail of protease and phosphatase inhibitors (#P002, NCM biotech) on ice for 30 min. Following this, the lysates were carefully resuspended and centrifuged at 10 000 × *g* for 10 min at 4 ℃. The corresponding lysates were mixed with 5 × SDS loading buffer and denatured at 95 ℃ for 5 min.

After denaturation, proteins were separated using SDS-PAGE and transferred onto PVDF membranes. The protein crosslinked membranes were then processed by blocking (Quickblock, #P0231, Beyotime) and incubated with the specified primary and secondary antibodies. Finally, the membranes were developed and visualised using an imaging system (Odyssey Sa, Li-Cor). The intensity of protein bands was quantified, taking into account the intensity of each sample in relation to its actin levels.

### Confocal assay

PAMs were seeded into glass-bottom dishes (CellVis) and cultured at 37 ℃ with 5% CO_2_ overnight to allow cell adherence. Following virus inoculation and/or treatment with chemicals or probes, the PAMs were fixed at RT for 30 min with 4% paraformaldehyde. Permeabilisation followed this with 0.1% TritonX-100 for 10 min and 5% BSA blocking for 30 min at RT. Both TritonX-100 and BSA were diluted and dissolved in 1 × PBS.

Next, the PAMs were incubated with the corresponding primary antibodies at 37 ℃ for 1 h, washed three times with 1 × PBS, and then incubated with the indicated secondary antibodies at 37 ℃ for another hour, followed by three additional washes with 1 × PBS. The cell nucleus was stained with DAPI (#P0131, Beyotime), and images were acquired using a confocal fluorescence microscope (TCS SP8, Leica). The average number of SG-positive PAMs was recorded in five random fields of view. The probe concentrations were determined according to the manufacturers’ instructions.

Additionally, because Thioltracker’s fluorescence excitation wavelength overlaps with that of DAPI, the Thioltracker images were directly captured via LAS X software, and the signal intensity was quantified using ImageJ.

### Virus titration (HAD assay)

The virus with an unknown titer was serially diluted tenfold in RPMI-1640 medium. These dilutions were used to inoculate PAMs in 96-well cell culture plates. Since the viral-encoded CD2v exhibits hemadsorption, 20 μL of a 1% porcine red blood cell suspension was added to each well after 12 hpi. Hemabsorption was observed under a microscope every 12 h up to 72 hpi. The final viral titers were calculated using the Reed-Muench method, with four biological replicates and four technical replicates for each dilution (4 × 4).

### Cell viability determination and iron assay

Cell viability was assessed after adding the corresponding chemicals using the Cell Counting Kit-8 kit (#FD3788, Fudebio-tech). The iron content was measured with an iron assay kit (#BC5415, Solarbio). All procedures were carried out according to the manufacturer’s instructions.

### Statistical analysis

The conventional Student *t*-test was conducted for all statistically significant calculations. The results are presented as the mean ± standard deviation from a minimum of three independent experiments. Five different eyesight fields were analysed for the calculations of SG-positive PAMs. A p-value of less than 0.05 (*p* < 0.05) was considered statistically significant, indicated with annotations as follows: *p < 0.05, ** p < 0.01, *** p < 0.001, and **** p < 0.0001.

## Results

### ASFV infection inhibits the formation of SGs, including those that are induced by Ars

**Figure 1:**
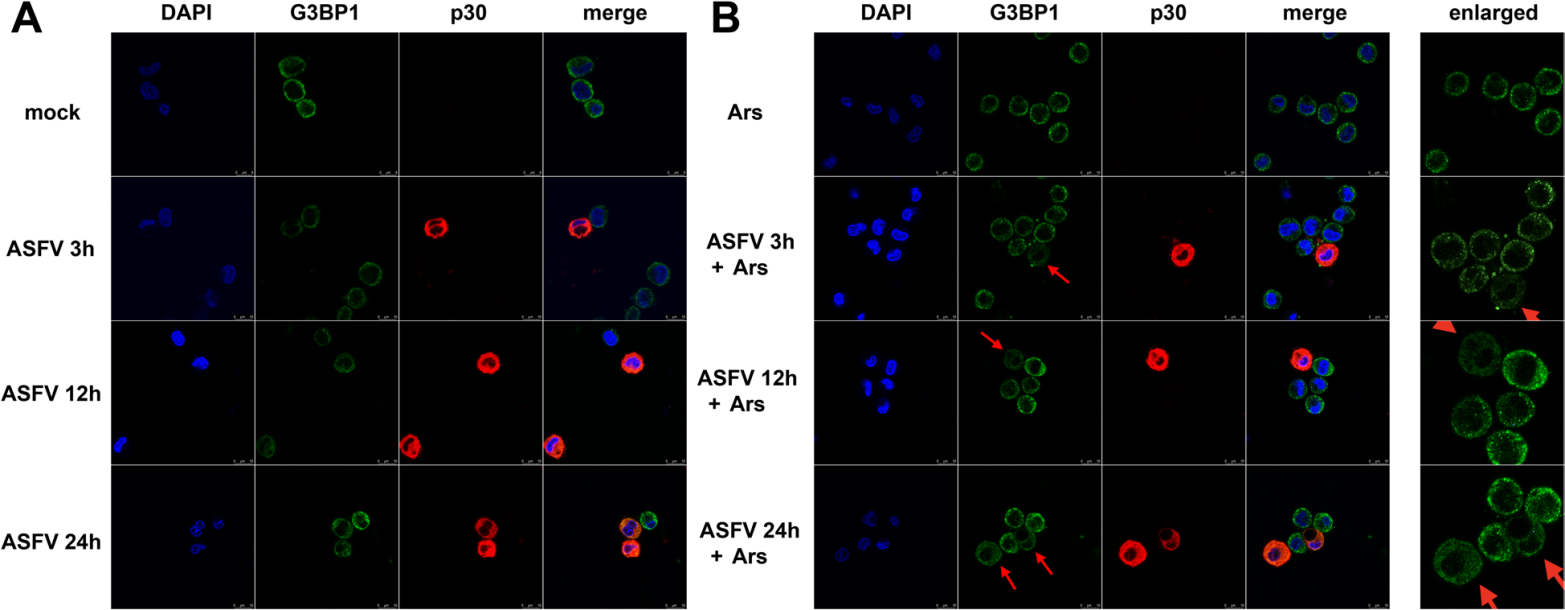
**ASFV inhibits SGs formation during infection**. (A) ASFV inhibits SGs formation at 3, 12, and 24 hpi (MOI = 1). (B) ASFV inhibits Ars-induced SGs formation at 3, 12, and 24 hpi (MOI = 1). Red arrows indicate SG-negative and ASFV-positive PAMs.

Oxidative stress was induced through treatment with Ars, leading to the activation of eIF2α kinases. This activation resulted in the phosphorylation of eIF2α, which triggered the formation of SGs. We first investigated whether ASFV infection prompted SGs formation. PAMs were infected ex vivo with the highly pathogenic genotype II strain, GZ201801_2, at an MOI of 1. We set three distinct time points—3 hpi, 12 hpi, and 24 hpi—to represent the early, middle, and late stages of ASFV infection. Interestingly, the ASFV-infected PAMs did not form SGs (Figure 1A).

Ars acts as an activator of ROS and can induce SGs [47, 48]. Therefore, before fixation, PAMs were treated with 0.5 mM Ars for 30 min. Notably, ASFV-infected PAMs inhibited Ars-induced SGs formation at 3, 12, and 24 hpi. These results suggest that ASFV has a solid ability to resist SGs formation (Figure 1B).

To determine whether this characteristic is universal among other ASFV strains, we used the genotype I ASFV strain, OURT88/3, to infect PAMs ex vivo at an MOI of 1. Consistent with the results from the ASFV GZ201801_2 strain, nearly all OURT88/3-infected PAMs did not show SGs formation under Ars induction, while almost 100% of the mock-infected PAMs treated with Ars contained SGs (Additional file 1A). Furthermore, the same assay performed with the three-large-fragment-deleted strain YNFN202103 showed consistent outcomes (Additional file 1B).

These findings demonstrated that various ASFV strains can inhibit Ars-induced SGs formation, indicating a generalised mechanism of inhibition in ASFV-infected PAMs that is not limited to specific ASFV strains.

The regulation of SGs by ADFV was investigated through the knockdown of G3BP1, a core component of SGs. Previous studies have demonstrated the potential antiviral role of SGs, and the knockdown of G3BP1 can inhibit the assembly of SGs. Consistent with earlier findings, ASFV infection significantly reduced the expression of pG3BP1 during the middle stage of infection (Additional file 1C).

In this study, PAMs were transfected with siRNAs targeting pG3BP1 before being infected with ASFV infection ex vivo (MOI = 1). The results showed that the level of ASFV *cp204l,* which encodes the ASFV p30 protein, increased when pG3BP1 was knocked down (Additional files 1D and 1E). Similarly, both the p30 protein level and the virus titer also increased at 12 hpi (Additional files 1F and 1G).

The lack of G3BP1 expression in si-NC transfected PAMs may result from the combined effects of G3BP1 downregulation by ASFV and si-G3BP1 transfection. However, the increased viral activity observed at 24 hpi may be influenced by other potential factors. Overall, these findings suggest that pG3BP1 negatively regulates ASFV transcription and replication, particularly during the early to middle stages of infection. Furthermore, the ASFV-mediated downregulation of G3BP1 and the inhibition of SGs formation may enhance viral replication and facilitate immune evasion.

### ASFV infection increases intracellular GSH levels

Previous studies have indicated that ASFV infection can induce ROS in cells [37, 49]. To investigate the levels of ROS in response to ASFV infection, we measured ROS levels in PAMs at 3 hpi, 12 hpi, and 24 hpi, comparing mock-infected and ASFV-infected groups. As expected, PAMs that were positive for the p30 protein exhibited a higher intensity of ROS signals than bystander PAMs and mock-infected PAMs, as demonstrated by probe-binding observations (Additional file 2).

**Figure 2:**
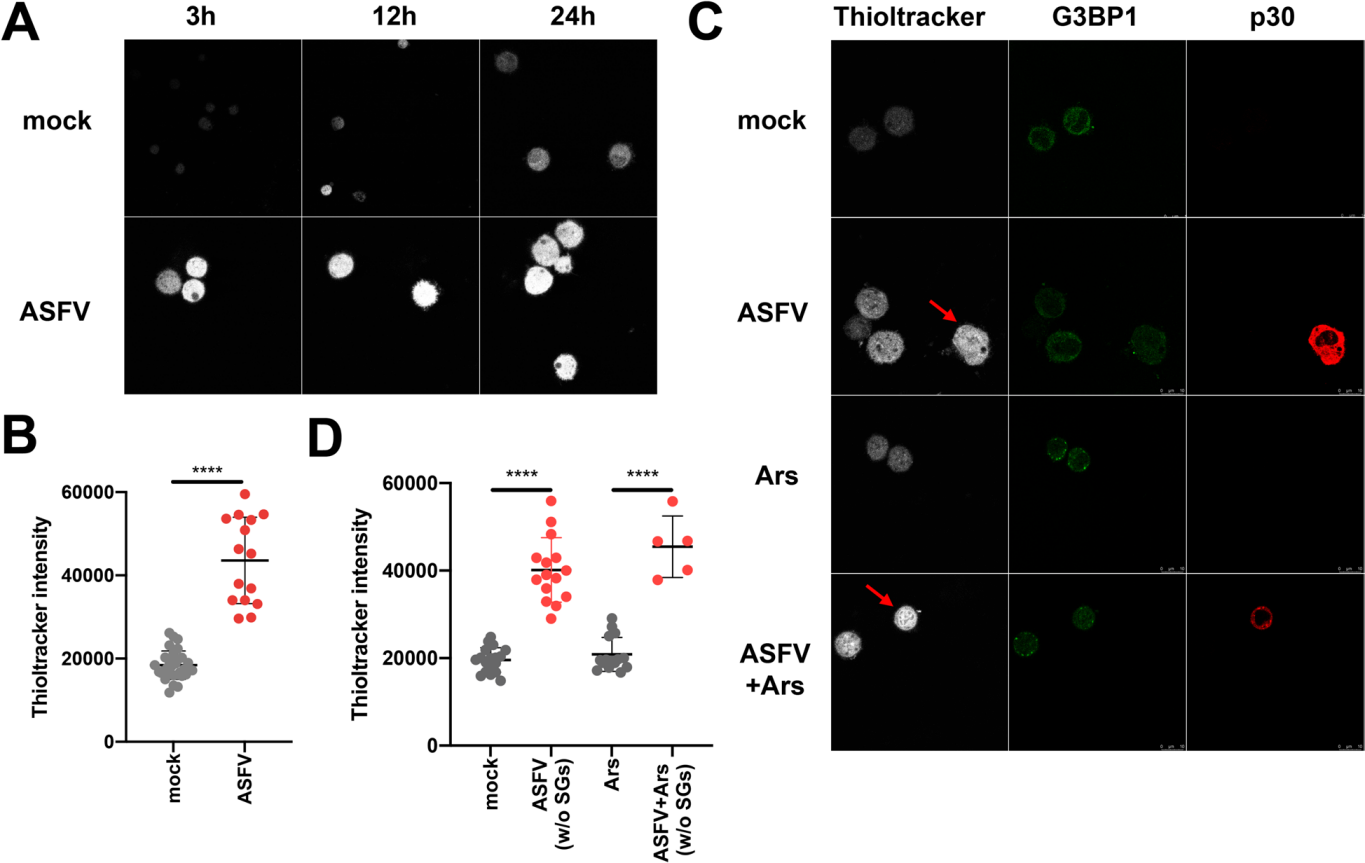
**Intracellular GSH levels were elevated following ASFV infection**. (A) Intracellular GSH levels were measured using specific probes, with signal intensity (white light) shown at 3, 12, and 24 hp with an MOI = 5. (B) Thioltracker signal intensity in individual PAMs was quantified and visualised using GraphPad Prism. Each dot represents the values for individual PAM. The longer horizontal lines indicate the mean values, while the error bars represent the standard deviation (SD). (C) PAMs were either mock-infected or ASFV-infected and treated with or without Ars at 12 hpi, with an MOI = 1. The SGs were detected using an anti-G3BP1 antibody, while intracellular GSH levels were labelled with the Thioltracker probe. Red arrows highlight that ASFV-infected PAMs exhibit higher GSH levels than mock-infected or Ars-treated PAMs. (D) GSH intensity was quantified and visualised.

GSH is one of the most abundant intracellular antioxidants, providing protection against various forms of oxidative stress by donating electrons to ROS. To assess whether intracellular GSH levels were altered by ASFV infection, we used GSH-specific probes on mock-infected and ASFV-infected PAMs. Preliminary findings showed that GSH signal intensity was consistently higher at different time points during ASFV infection compared to the mock-infected group (Figures 2A and B).

Furthermore, we investigated the upregulation of intracellular GSH and its relationship with intracellular Sgs. ASFV-infected PAMs at 24 hpi and MOI = 1 demonstrated a significantly higher GSH signal compared to both mock-infected and bystander PAMs, regardless of Ars treatment. Importantly, the formation of SGs induced by Ars did not change substantially intracellular GSH production (Figures 2C and D). Additionally, nearly all PAMs produced SGs after Ars treatment before harvesting. Consistent results were observed when we quantified intracellular GSH levels at the individual cell level (Figures 2B and D). We further elucidated the role of GSH upregulation in ASFV’s resistance to intracellular SGs formation as a mechanism for immune evasion.

### The impact of depleting or supplying intracellular GSH levels on ASFV-mediated PAMs resistance to Ars-induced SGs formation

L-Buthionine-(S,R)-sulfoximine (BSO) is a potent and irreversible inhibitor of γ-glutamylcysteine synthetase, an essential enzyme involved in GSH synthesis. This inhibition leads to a depletion of intracellular GSH levels [50–52].

To assess the viability of PAMs, experiments were conducted after 48 h treatment with BSO (Additional file 3A). PAMs were cultured in the presence of absence of 5 mM BSO for 24 h and then mock-infected or infected with ASFV ex vivo at an MOI of 1. The concentration of BSO was maintained throughout the infection period.

Importantly, over 70% of the mock-infected PAMs treated with BSO formed SGs, indicating that BSO-induced reduction in intracellular GSH levels promotes SGs formation by disrupting redox homeostasis. In contrast, the percentage of SG-positive PAMs that were ASFV-infected increased from less than 10% without BSO treatment to approximately 50% with BSO treatment at 24 hpi and MOI = 1. These results suggest that the impairment of intracellular GSH synthesis by BSO reduces the ability of ASFV-infected PAMs to manage elevated levels of ROS, thereby diminishing the inhibitory effect on SGs formation.

**Figure 3:**
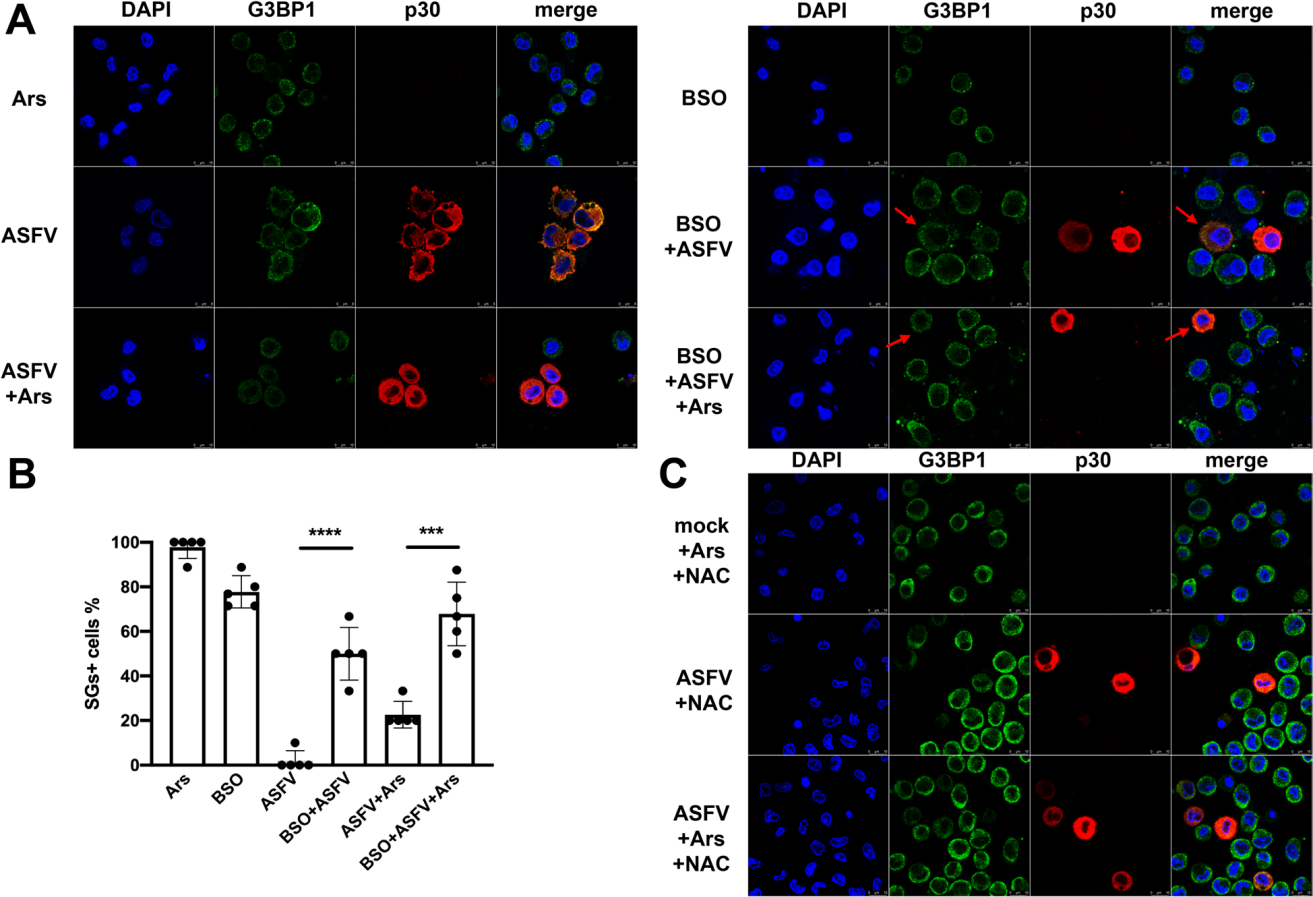
**Effect of depleting or supplying GSH on ASFV-mediated (MOI = 1) PAMs resistance to Ars-induced SGs formation at 12 hpi.** (A) PAMs were mock- or ASFV-infected and treated with PBS, Ars, BSO, or Ars and BSO. The impact of BSO-mediated GSH depletion on SGs formation. Red arrows indicate SGs formation in ASFV-infected PAMs under BSO treatment. (B) The number of SG-positive, mock-infected, or ASFV-infected PAMs was recorded and quantified from at least five fields for each of the three biological repeats. (C) PAMs were mock-infected or ASFV-infected. PAMs were also treated with NAC (10 mM) with or without Ars (0.5 mM) and then fixed and processed for IFA.

Further confirmation of these findings was obtained through treatment with Ars. Almost all mock-infected PAMs treated with Ars formed SGs, whereas ASFV-infected PAMs showed minimal SGs formation, even after Ars treatment [32, 33]. Additionally, PAMs that were either mock- or ASFV-infected and subsequently treated with Ars demonstrated a significant increase in the proportion of SG-positive PAMs in the BSO treatment group compared to the non-BSO treatment group. This increase was observed in both ASFV-infected PAMs and Ars-treated ASFV-infected PAMs (Figures 3A and B). These results suggest that stable GSH synthesis is crucial for inhibiting SGs formation by maintaining a balanced intracellular redox balance.

N-acetylcysteine (NAC), recognised for its ability to inhibit ROS and serve as a precursor to GSH, has also been shown to act as a GSH activator [45, 53]. When applied to cells, NAC can enhance intracellular GSH levels, protecting against damage caused by oxidative stress.

In this section, the viability of PAMs under NAC treatment was first assessed using the CCK-8 assay (Additional file 3B). NAC (10 mM) was administered to elevate intracellular GSH levels in PAMs. Both mock-infected and ASFV-infected PAMs were treated with NAC alone or in combination with Ars for 1 h at 24 hpi, followed by fixation for a confocal assay. The NAC-treated PAMs, regardless of being mock-infected or ASFV-infected and whether treated with Ars or not, showed almost no formation of SGs (Figure 3C). These findings suggest that elevated intracellular GSH levels can inhibit the formation of Ars-induced SGs. Additionally, the upregulation of GSH levels during ASFV infection is crucial for suppressing SGs formation in PAMs, including those induced by Ars.

### ASFV-regulated increase of intracellular GSH levels also inhibits ferroptosis

**Figure 4:**
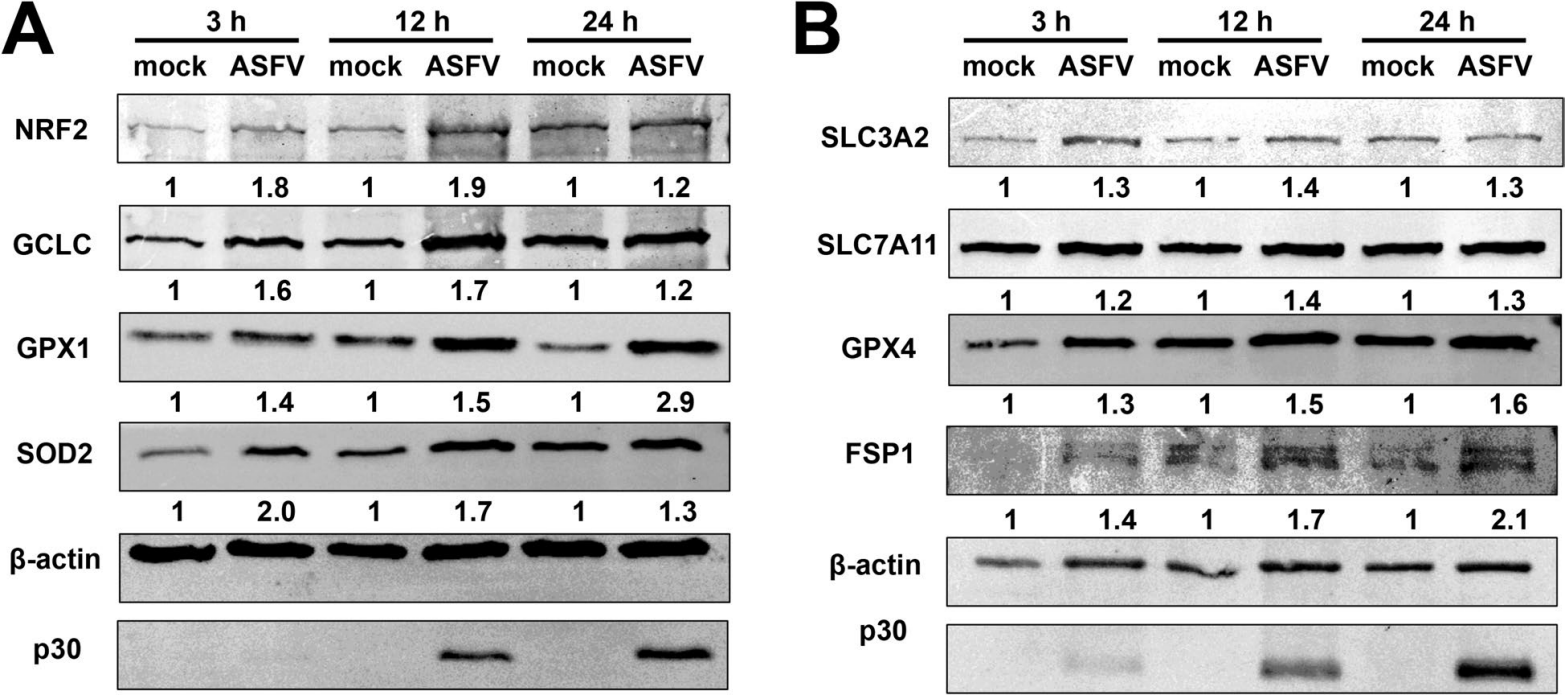
**Indicated GSH-related factors determination of mock- and ASFV-infected PAMs (MOI = 1).** Lysates harvested from mock-infected or ASFV-infected PAMs were analysed at various times post-infection to determine levels of GSH-related factors. (A) NRF2, the GSH synthesis and regeneration pathways related factors, and (B) GSH-associated ferroptosis related factors via immunoblotting.

After infecting PAMS ex vivo with ASFV at MOI = 1, we observed a rapid increase in GSH levels (Figures 2A and C). The nuclear factor erythroid 2-related factor (NRF2), a crucial transcription factor responsible for regulating various antioxidative genes involved in GSH synthesis and regeneration, was assessed at both the mRNA and protein levels. Results indicated a significant upregulation of NRF2 during ASFV infection (Figure 4A and Additional file 4A).

To explore the upregulation of downstream antioxidative pathway-related genes, we evaluated the protein levels of antioxidative gene products in both mock-infected and ASFV-infected PAMs at various time points. The results showed a notable increase in the levels of the glutamate-cysteine ligase catalytic subunit (GCLC), glutathione peroxidase 1 (GPX1), and superoxide dismutase 2 (SOD2) at 3, 12, and 24 hpi (Figure 4A). The increase in intracellular GSH is also associated with the inhibition of ferroptosis.

Consequently, we examined several key factors in the ferroptosis-related pathway involved in GSH synthesis and transport in both mock and ASFV-infected PAMs. We observed upregulation of the two subunits of the Xc^−^ system, SLC3A2 and SLC7A11 (Figure 4B). SLC3A2 showed a significant increase in expression during ASFV infection, while SLC7A11 exhibited only a slight upregulation. Additionally, glutathione peroxidase 4 (GPX4) levels were markedly elevated.

Furthermore, FSP1, an important factor in the Xc^−^ system/GPX4-independent ferroptosis pathway, demonstrated increased levels at various time points in ASFV-infected PAMs. This suggests that FSP1, GSH, and GPX4 work synergistically to inhibit lipid peroxidation while maintaining metabolic homeostasis at the cellular level (Figure 4B).

To further investigate whether ASFV can inhibit ferroptosis, we conducted an iron assay using BSO and the ferroptosis activator Erastin (Additional files 4B and C). The results indicated that ASFV infection countered the ferroptosis induced by either Erastin or BSO, as evidenced by the relative levels of Fe^2+^. These findings highlight the upregulation of antioxidative enzymes involved in GSH synthesis and regeneration during ASFV infection and suggest that GSH plays a critical role in maintaining metabolic homeostasis, evading the immune response, and supporting viral replication.

### ASFV increases intracellular GSH levels by upregulating mitochondrial pAIFM1, which in turn inhibits SGs formation

The pAIFM1 protein, a homolog of FSP1(AIFM2), plays a crucial role in modulating cellular apoptosis and regulating redox homeostasis. It has been reported to be potentially expressed in mitochondria. In this study, we conducted co-localisation experiments using the specific mitochondrial probe Mito-Red and an antibody against pAIFM1 to determine its subcellular localisation. The results indicated that pAIFM1 was highly co-localised with Mito-Red, regardless of whether cells were mock-treated or infected with ASFV, thereby confirming pAIFM1’s mitochondrial subcellular localisation (Additional file 4D).

**Figure 5:**
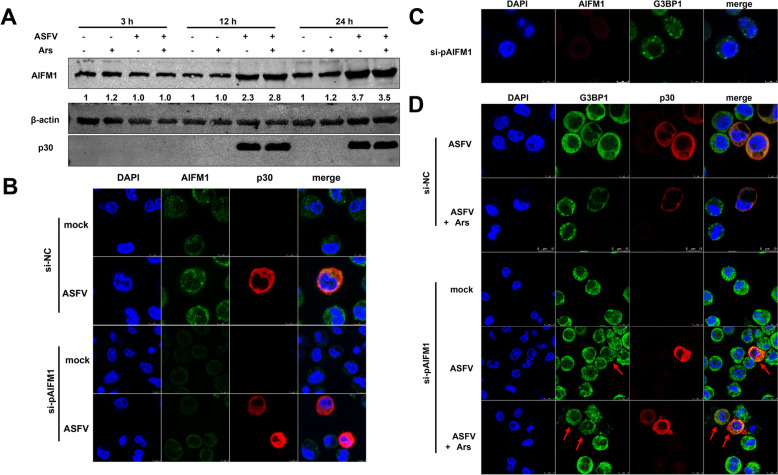
**AIFM1 is an important target for regulating the intracellular antioxidant capacity of ASFV-infected PAMs (MOI = 1).** (A) Lysates were harvested from PAMs infected with PBS or ASFV and treated with PBS or Ars, followed by WB at 24 hpi. (B) PAMs were transfected with si-AIFM1 for AIFM1-knockdown and followed by mock- or ASFV-infection. Staining with anti-AIFM1 (green) and anti-p30 (red) was used to visualise the results. (C) SGs formation in AIFM1-knockdown PAMs, with staining using anti-AIFM1 (red) and anti-G3BP1 (green). (D) PAMs were transfected with si-NC or si-AIFM1 for 24 h followed by mock- or ASFV-infection. PAMs were treated with Ars or PBS at 24 hpi followed by a confocal assay using anti-G3BP1 (green) and anti-p30 (red). Red arrows indicate obvious SGs formation in ASFV-infected PAMs under AIFM1-knockdown.

Subsequently, PAMs were either mock-infected or infected with ASFV (MOI = 1) and treated with PBS or Ars for 30 min before harvesting at 3, 12, or 24 hpi. The results showed that treatment with Ars did not significantly affect pAIFM1 levels. In contrast, ASFV infection significantly upregulated the protein level of pAIFM1, particularly during the middle and late stages of infection, highlighting the unique role of ASFV in promoting pAIFM1 expression (Figures 5A and B).

Next, we examined the effects of pAIFM1 knockdown on intracellular redox levels and the formation of SGs. Effective knockdown of intracellular pAIFM1 was first observed following transfection with si-pAIFM1 (Figure 5B). Additionally, AIFM1-knockdown induced the formation of intracellular SGs, suggesting that the regulation of GSH levels by pAIFM1-was disrupted, leading to intracellular SGs formation (Figure 5C). Following pAIFM1-knockdown, ASFV infection also resulted in SGs formation, including ASFV-positive PAMs (Figure 5D). This indicates that pAIFM1-knockdown significantly weakens the capacity of ASFV to inhibit SGs formation. Therefore, pAIFM1 is a critical target for maintaining redox balance and regulating intracellular GSH levels during ASFV infection.

### Impact of depleting or supplying intracellular GSH on ASFV replication

**Figure 6:**
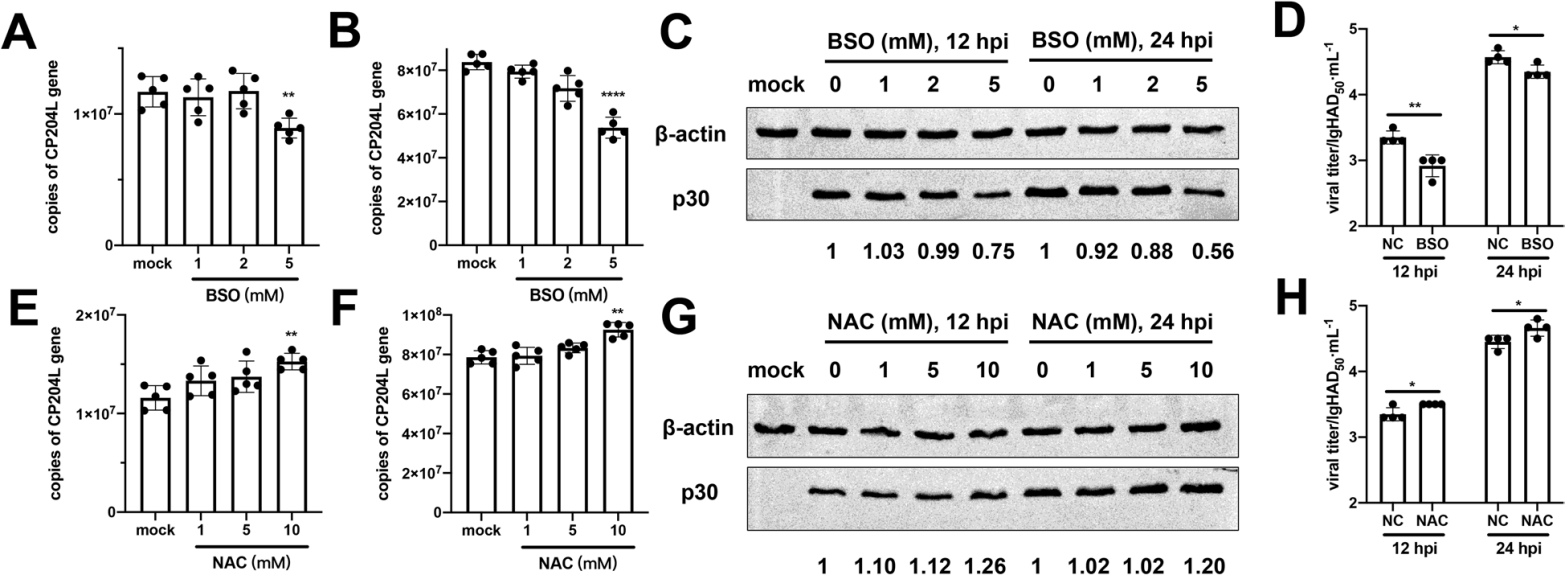
**Impact of GSH depletion or supplementation on ASFV replication in PAMs (MOI = 1)**. (A and B) *cp204l* levels, (C) p30 levels, and (D) viral titers at 12 and 24 hpi under various concentrations of BSO treatments. (E and F) *cp204l* levels, (G) p30 levels, and (H) viral titers at 12 and 24 hpi under various concentrations of NAC treatments.

BSO and NAC were used in this study as GSH inhibitors and activators to investigate the effects of depleting or replenishing intracellular GSH on the formation of SGs. PAMs were pretreated with various concentrations of BSO for 24 h before being infected with ASFV (MOI = 1) ex vivo. Samples were collected at 12 and 24 hpi, with the BSO concentrations maintained throughout the experiment. The results demonstrated a significant decrease in the copy number of *cp204l* and the levels of the p30 protein following treatment with 5 mM BSO at both time points (Figures 6A–C). Furthermore, the viral titers in PAMS treated with 5 mM BSO were significantly lower at both 12 and 24 hpi compared to those in the mock-treated PAMs (Figure 6D).

In contrast, PAMs were pretreated with various concentrations of NAC for 12 h before ASFV infection. The results indicated that 10 mM NAC significantly increased the *cp204l* copy number and p30 protein levels (Figures 6E–G). PAMS infected with ASFV and treated with NAC exhibited higher viral titers than the mock-treated PAMs (Figure 6H). Further analysis involving AIFM1 knockdown showed reduced viral replication, suggesting that intracellular GSH levels are essential for ASFV replication (Additional file 4G).

## Discussion

SGs are non-membranous cytoplasmic structures that form within eukaryotic cells in response to various stressors, including heat shock, oxidative stress, ultraviolet radiation, and viral infections. These granules primarily consist of stalled translation initiation complexes on mRNAs and a variety of RNA-binding proteins. The formation of SGs is a part of the cellular response to external stress, acting as a temporary suppressor of mRNA translation. This function supports the modulation of stress responses, helps mitigate stress-induced damage, and typically allows for the resumption of normal protein synthesis once the stress has subsided. SGs are also thought to have antiviral properties.

ASFV is an arthropod-borne DNA virus with a large viral genome that poses a significant risk to pig health, resulting in substantial economic losses for the swine industry and related sectors. Despite this serious impact, our understanding of ASFV still needs to be improved. Previous studies have identified various mechanisms by which ASFV counteracts the formation of SGs. These include the ASFV-specific cleavage of G3BP1 by the virus-encoded protein pS273R, as well as the counteraction of SGs formation through both DP71L-dependent and DP71L-independent dephosphorylation of eIF2α [29–33].

Our research has uncovered an additional eIF2α-independent pathway by which ASFV inhibits SGs formation. These findings indicate that ASFV infection in PAMs in vitro increases intracellular GSH levels, which helps protect ASFV-infected PAMs from excessive oxidative stress. This upregulation also counteracts the formulation of cellular SGs, creating a more favourable environment for viral replication.

Confocal assays confirmed that ASFV infection does not lead to the formation of SGs and actually suppresses the formation of SGs induced by Ars. This finding is consistent with previous studies [32, 33, 54, 55]. However, the mechanisms behind this phenomenon remain unclear. It has been reported that ASFV infection can trigger cellular ROS, which typically promotes SGs formation [37, 47, 56]. This relationship leads to the following hypothesis: Does ASFV infection involve a mechanism that prompts cellular antioxidants to neutralise intracellular ROS accumulation? If so, this could explain the ASFV-induced suppression of SGs formation, facilitating viral replication and helping the virus evade the immune response.

Under normal conditions, various metabolic reactions occur within cellular structures such as mitochondria, peroxisomes, and the endoplasmic reticulum. These processes can lead to the continuous generation of low-level intracellular ROS, which have positive functions [57].

Notably, RNA viruses can alter the antioxidant system during viral infection. For example, infections with influenza or hepatitis C viruses can trigger the production of high levels of ROS while simultaneously downregulating intracellular GSH levels [58–61]. In contrast, our study revealed that ASFV can induce ROS activation alongside an increase in intracellular GSH levels.

The simultaneous upregulation of both ROS and GSH is not contradictory, as similar patterns have been observed in tumour cells [62]. Elevated levels of ROS and GSH are necessary to maintain homeostasis in these cells. This was evidenced by the enhanced intensity of GSH signals detected with the Thioltracker GSH probe in ASFV-infected cells and the upregulation of various components of antioxidant pathways, such as GCLC. Additionally, the key transcription factor NRF2, which regulates oxidative stress and antioxidant homeostasis, was also found to be upregulated following ASFV infection.

Consistent with our findings, infections by KHSV and HCMV have also been reported to lead to the upregulation of NRF2 [58, 63]. While it is commonly claimed that viral infections can cause NRF2 upregulation, this does not necessarily imply that intracellular GSH levels are also increased. In fact, evidence suggests that NRF2 upregulation may primarily represent a potential resistance mechanism against ROS [64].

For example, a study on HCV infection showed NRF2 induction during the initial stages of infection; however, GSH levels remained stable during this period, indicating that the cells had sufficient reductive capacity at that time. In contrast, GSH depletion has been observed during the middle to late stages of viral infection [64–66].

Given this interesting phenomenon, our study aimed to validate the key factors involved in the GSH synthesis and regeneration pathways during ASFV infection. We also investigated the GSH-related ferroptosis pathway, revealing that ASFV can upregulate essential factors in these pathways, such as GCLC, SLC7A11/SLC3A2, SOD2, and FSP1, which help preserve intracellular redox homeostasis [67].

Research using single-cell RNA-seq metadata suggests that some of these factors are upregulated during ASFV infection [68]. Studies on various viruses indicate that many can trigger ferroptosis, while HBV has been shown to inhibit ferroptosis to enhance its replication [69, 70].

Using erastin and BSO provides a means to assess whether ASFV infection reduces ferroptosis induced by these drugs. Iron assays showed similar results for ASFV and HBV, indicating that the upregulation of intracellular GSH caused by ASFV is a sophisticated mechanism that benefits the replication environment of ASFV.

This study used the GSH inhibitor BSO and the activator NAC to examine the relationship between ASFV infection, intracellular GSH levels, and the formation of SGs. Our results showed that the upregulation of GSH levels induced by ASFV infection contributed to the suppression of SGs formation. Modulating GSH levels, either by depleting or supplying it, can also influence ASFV replication. These findings confirm that the ASFV-induced increase of GSH creates a more favourable intracellular environment for viral replication by suppressing SGs, which helps the virus evade immune responses.

The treatment of AIFM1-knockdown HeLa cells can significantly reduce the oxidation of NAD(P)H and the levels of intracellular GSH, leading to an increase in the number of cells positive for Ars-induced SGs [45, 71]. In this context, our study using si-pAIFM1 transfection, western blotting, and confocal microscopy revealed that ASFV can elevate intracellular GSH levels by targeting the mitochondrial pAIFM1. The expression of pAIFM1 is upregulated following ASFV infection, regardless of Ars treatment.

AIFM1 plays a crucial role in maintaining steady levels of intracellular GSH. The knockdown of endogenous pAIFM1 in PAMs significantly increased the formation of intracellular SGs, irrespective of ASFV infection status. Interestingly, AIFM1 can also induce cellular apoptosis, although research suggested that AIFM1’s regulation of SGs is distinct from its role in inducing cellular apoptosis; however, research indicates that its regulation of SGs operates independently of its role in promoting apoptosis.

Consistent with previous studies, ASFV is known to suppress cellular apoptosis and other life-dependent processes during the early phases of infection while later activating these processes in the middle to late stages [68, 71]. Additionally, our study investigated whether ASFV infection could modulate the expression levels of SLC25A39 and SLC25A40, both of which are known to stabilise intracellular GSH and maintain redox balance by influencing mitochondrial GSH levels [72–74]. Our experimental findings demonstrated that the expression levels of these two molecules remained stable following ASFV infection (data not shown).

Given the critical role that mitochondrial GSH plays in regulating cellular life processes, it is essential to comprehensively explore its function during ASFV infection. Furthermore, while it has been reported that HCV’s NS5a helps infected cells manage oxidative stress by regulating GPX4, this effect was not observed at the virus level [66]. Identifying ASFV-encoded genes responsible for mediating GSH upregulation could provide deeper insights into ASFV pathogenesis and potential therapeutic targets.

ASFV employs multiple mechanisms to interfere with the formation of SGs. For instance, ASFV-encoded pS273R specifically cleaves G3BP1, and ASFV also reprograms GSH levels, as previously detailed [33]. However, the exact mechanisms through which ASFV inhibits SGs formation still need to be fully understood. Our earlier experimental results showed that ASFV can inhibit SGs induced by heat stress, specifically when the cells were exposed to 42 ℃ for 45 min before harvest (data not shown). This finding is consistent with the virus’ ability to inhibit SGs induced by Ars. Given the crucial role of ubiquitin-dependent degradation in SGs formation during heat stress, exploring whether ASFV alters this ubiquitin-dependent degradation in SGs could be a promising avenue for further research [75].

**Figure 7:**
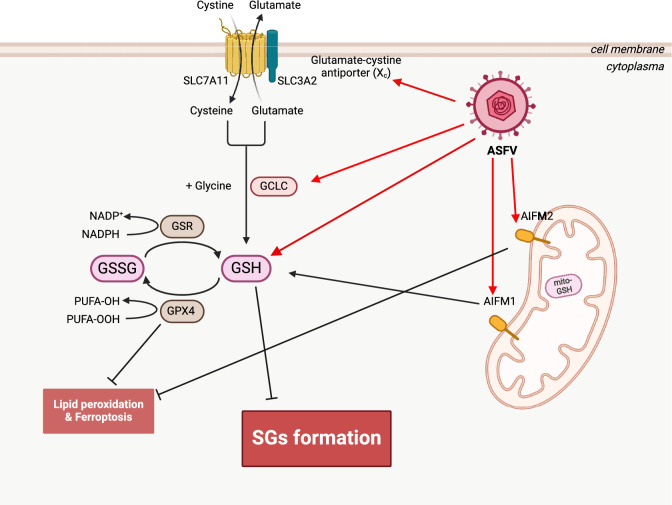
**The schematic diagram of the mechanism by which ASFV elevates intracellular GSH levels through multiple pathways, thereby antagonising the intracellular SGs formation and creating an optimised viral replication environment** (created with Biorender).

Infection with ASFV can increase intracellular ROS levels and activate the key antioxidant transcription factor, NRF2. Moreover, ASFV infection promotes the upregulation of various antioxidants involved in the synthesis and regeneration of GSH and the GSH-related ferroptosis pathways. Additionally, ASFV infection enhances mitochondrial levels of pAIFM1 (Figure 7). These critical factors help maintain elevated GSH levels in PAMs during viral infection and modulate the antioxidant system.

The regulation of these components by ASFV creates a unique intracellular antioxidative balance. Although ASFV leads to a significant accumulation of ROS, there is sufficient neutralisation by GSH. This process helps prevent the formulation of intracellular SGs, ultimately creating a more favourable environment for viral replication.

Our research offers a new perspective by suggesting that ASFV not only upregulates GSH, inhibiting the formation of SGs (including those induced by Ars) but also enhances conditions for ASFV replication within PAMs. In conclusion, this study offers fresh insights into the pathogenic mechanisms of ASFV and improves our understanding of its behaviour within host cells.

## Supplementary Information

**Additional file 1. The regulation of overexpression or knockdown of G3BP1 on ASFV replication**. (A) PAMs were mock- or ASFV-infected (genotype I strain OURT88/3) for 24 h followed by Ars treatment. Red arrow indicates the absence of SGs in ASFV-infected PAMs. (B) Lysates were harvested from mock- or ASFV-infected PAMs at different times after infection followed by immunoblotting using anti-G3BP1. (C) Besides representative genotype II strain GZ201801_2, a characteristic gene-deleted strain (YNFN202103) also inhibits Ars-induced SGs formation at 24 hpi. (D to G) PAMs were subjected to G3BP1-knockdown via si-RNA transfection, followed by ASFV inoculation. (D and E) The copies number of the *cp204l* gene at 12 and 24 hpi were determined. (F) The protein levels of the viral p30 were determined. (G) The viral titres were determined. The red arrows in 1C indicate SG-negative and ASFV-positive PAMs.

**Additional file 2. ASFV induces intracellular ROS production**.

**Additional file 3. Cell viability and working concentrations of BSO and NAC**.

**Additional file 4. The relationship between ASFV with Fe**^2+^ **levels or AIFM1 levels**. (A) The mRNA levels of *nrf2* in PAM infected with or without ASFV at various time points after infection. (B & C) ASFV (MOI = 1) can counteract Erastin- (15 μM) and BSO-induced upregulation of Fe^2+^ levels. (D) Co-localization assay of AIFM1 and mitochondria using probe Mito-Red (red) and anti-AIFM1 antibody (green). (E) si-AIFM1 knockdown can downregulate intracellular GSH levels. (F) The AIFM1-knockdown was evaluated under si-AIFM1 transfection. (G) AIFM1 knockdown inhibits ASFV replication (MOI = 1).

## Data Availability

The authors confirm that the data supporting this study's findings are available in the article and its supplementary materials.
