# Supplementary figures and images for: African swine fever virus enhances viral replication by increasing intracellular reduced glutathione levels, which suppresses stress granule formation
Source: Vet Res. 2024 Dec 20;55:172. doi: 10.1186/s13567-024-01433-4 (PMC11662820; doi:10.1186/s13567-024-01433-4)

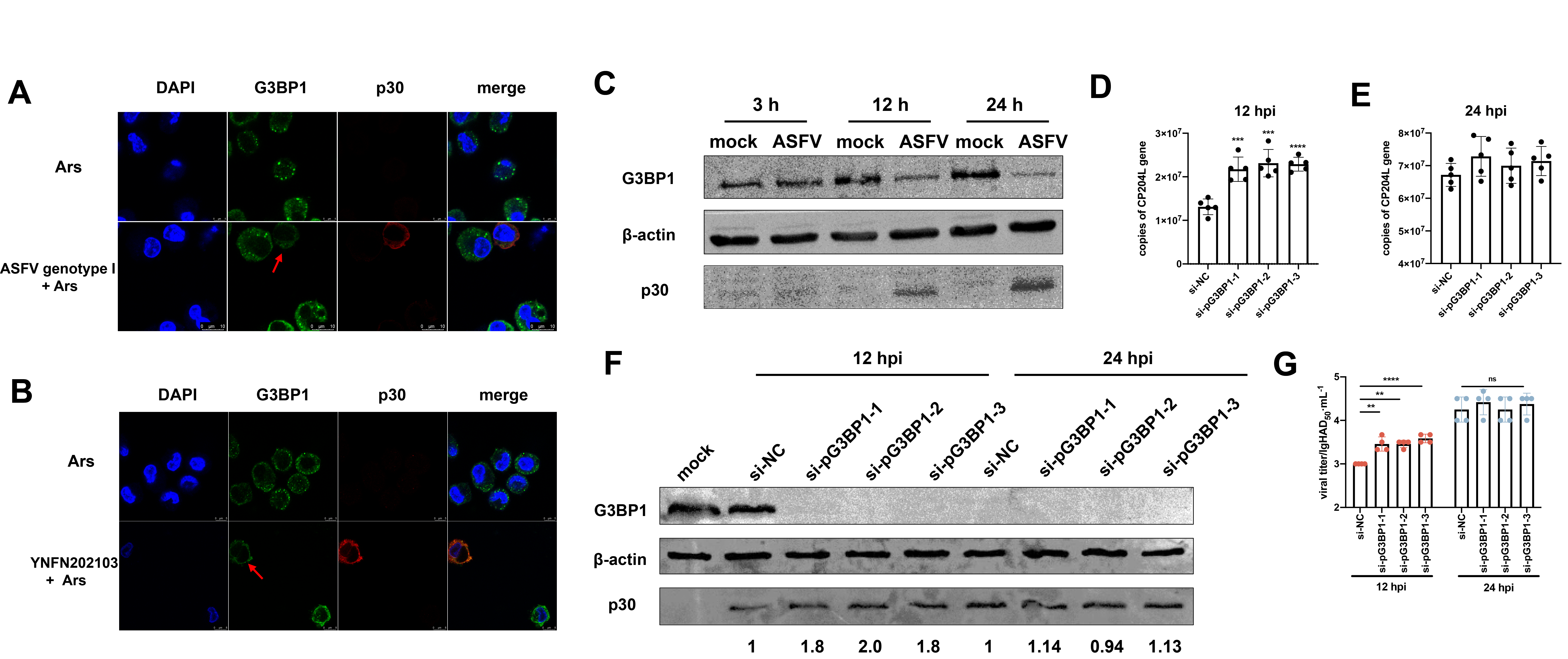

Supplement: Supplementary file 1 — Additional file 1. The regulation of overexpression or knockdown of G3BP1 on ASFV replication. (A) PAMs were mock- or ASFV-infected (genotype I strain OURT88/3) for 24 h followed by Ars treatment. Red arrow indicates the absence of SGs in ASFV-infected PAMs. (B) Lysates were harvested from mock- or ASFV-infected PAMs at different times after infection followed by immunoblotting using anti-G3BP1. (C) Besides representative genotype II strain GZ201801_2, a characteristic gene-deleted strain (YNFN202103) also inhibits Ars-induced SGs formation at 24 hpi. (D to G) PAMs were subjected to G3BP1-knockdown via si-RNA transfection, followed by ASFV inoculation. (D and E) The copies number of the cp204l gene at 12 and 24 hpi were determined. (F) The protein levels of the viral p30 were determined. (G) The viral titres were determined. The red arrows in 1C indicate SG-negative and ASFV-positive PAMs. [file 13567_2024_1433_MOESM1_ESM.png]

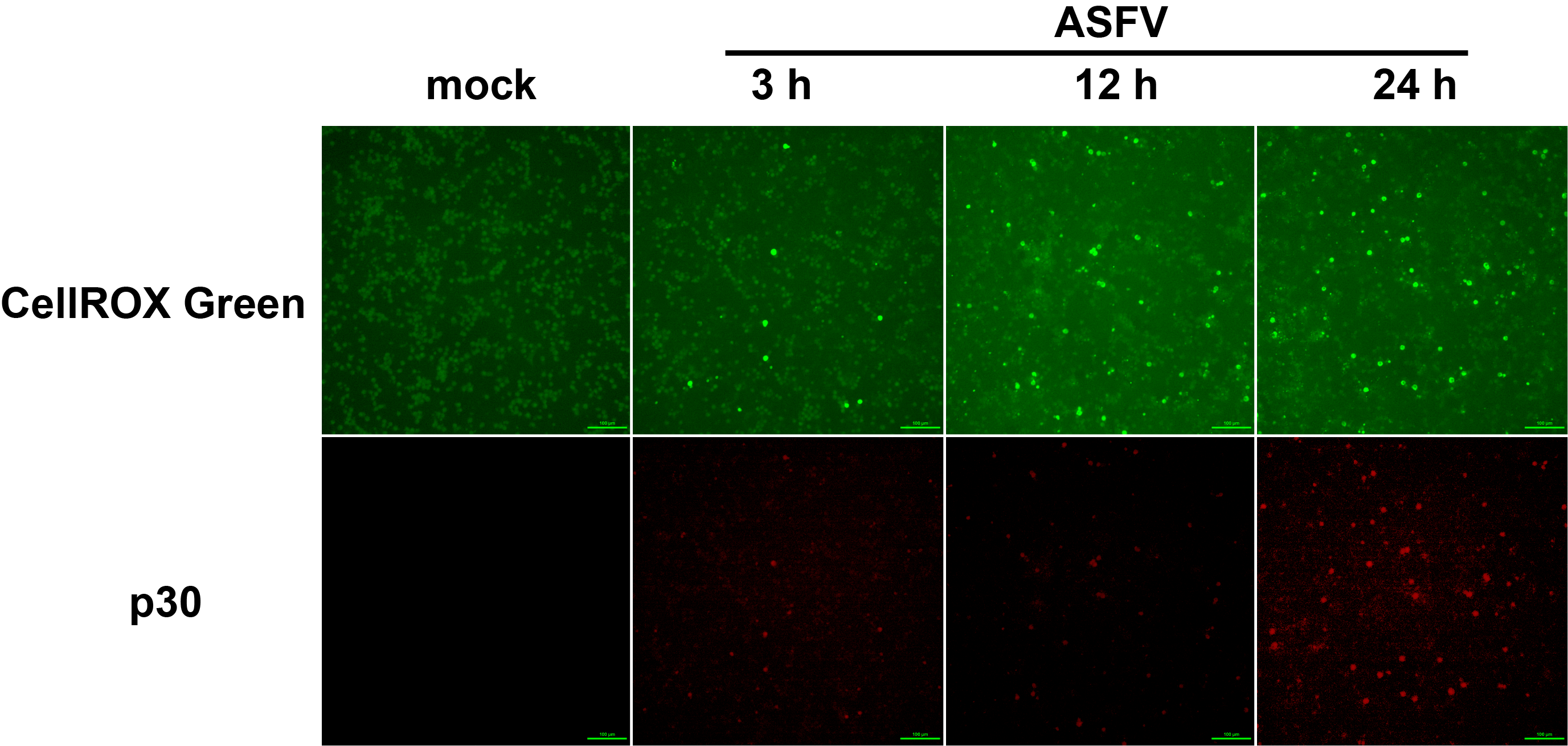

Supplement: Supplementary file 2 — Additional file 2. ASFV induces intracellular ROS production. [file 13567_2024_1433_MOESM2_ESM.png]

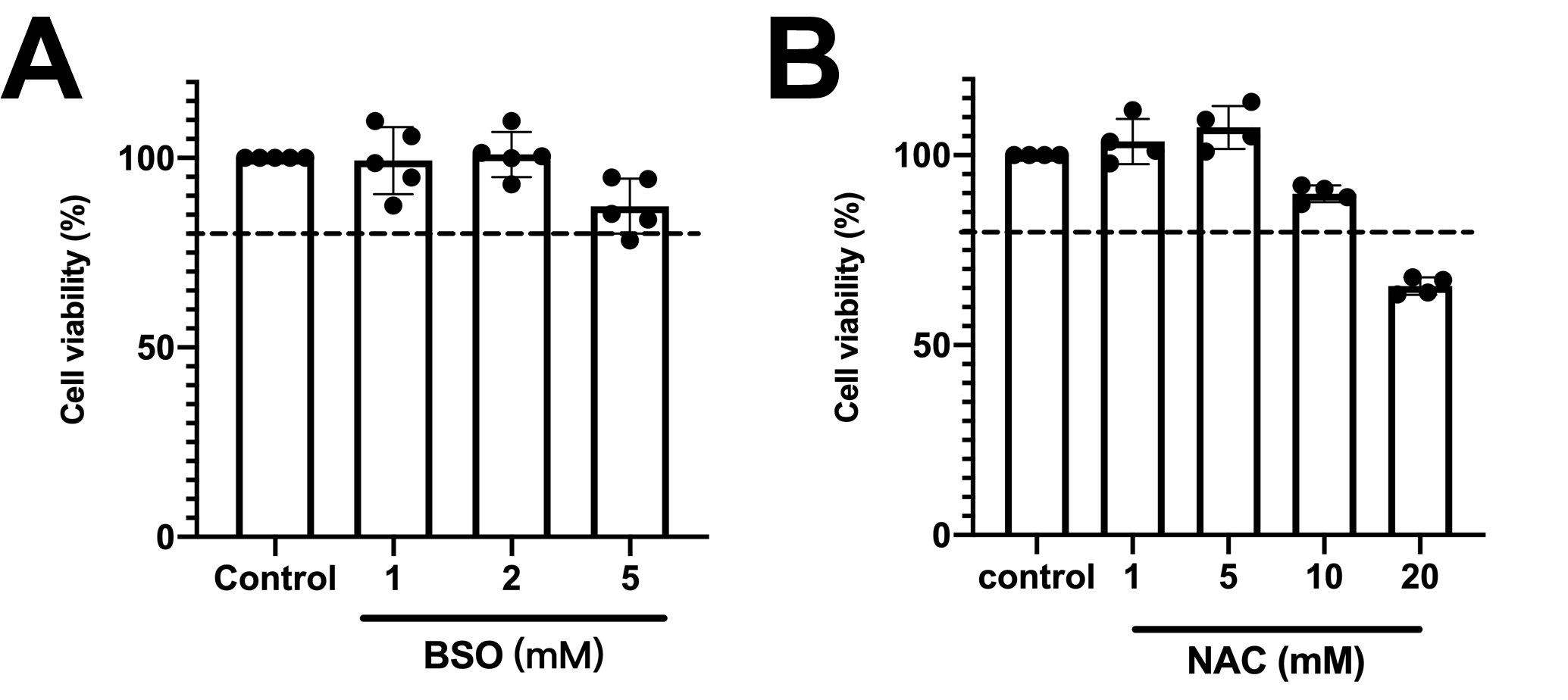

Supplement: Supplementary file 3 — Additional file 3. Cell viability and working concentrations of BSO and NAC. [file 13567_2024_1433_MOESM3_ESM.png]

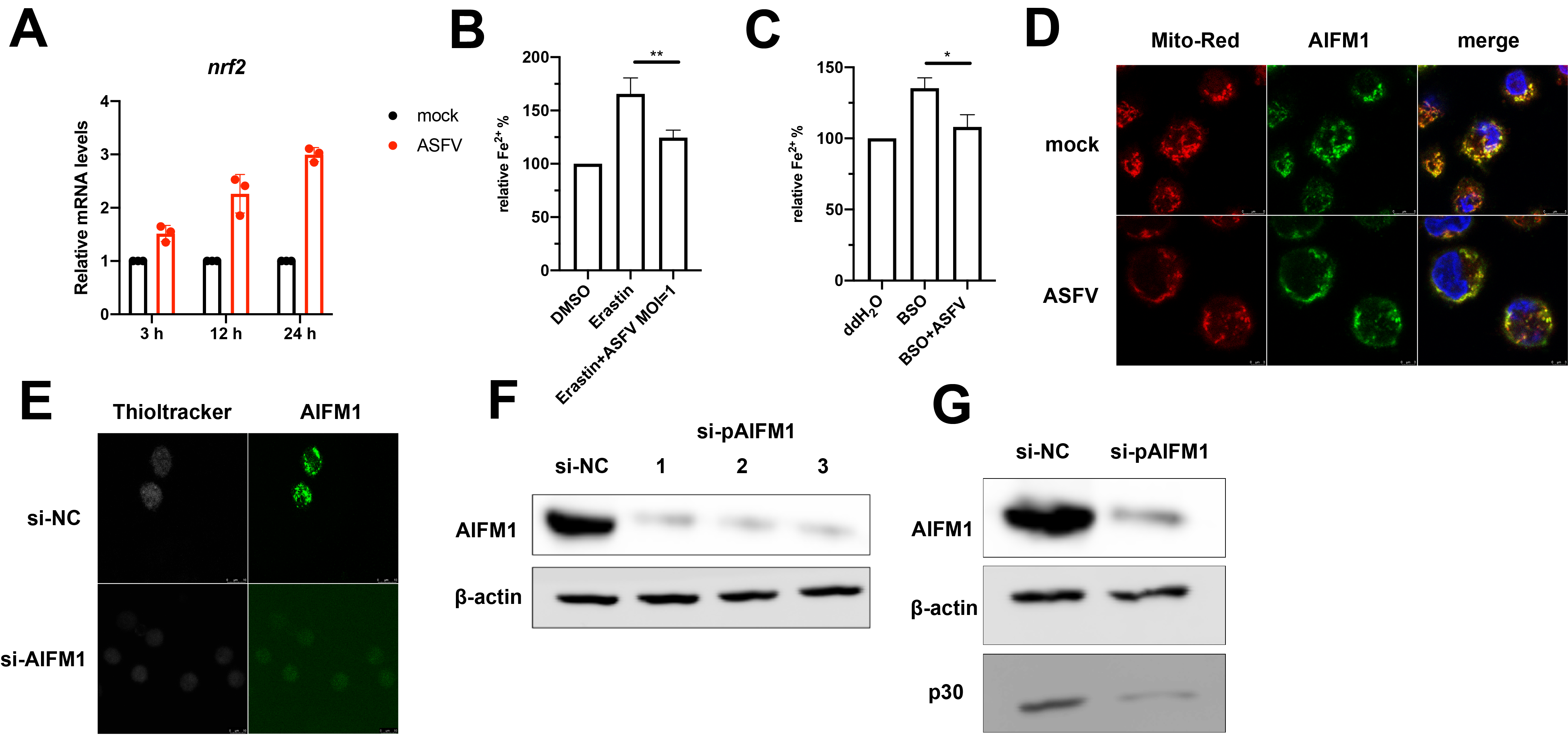

Supplement: Supplementary file 4 — Additional file 4. The relationship between ASFV with Fe2+ levels or AIFM1 levels. (A) The mRNA levels of nrf2 in PAM infected with or without ASFV at various time points after infection. (B & C) ASFV (MOI = 1) can counteract Erastin- (15 μM) and BSO-induced upregulation of Fe2+ levels. (D) Co-localization assay of AIFM1 and mitochondria using probe Mito-Red (red) and anti-AIFM1 antibody (green). (E) si-AIFM1 knockdown can downregulate intracellular GSH levels. (F) The AIFM1-knockdown was evaluated under si-AIFM1 transfection. (G) AIFM1 knockdown inhibits ASFV replication (MOI = 1). [file 13567_2024_1433_MOESM4_ESM.png]
